# Prehypertension Tsunami: A Decade Follow-Up of an Iranian Adult Population

**DOI:** 10.1371/journal.pone.0139412

**Published:** 2015-10-06

**Authors:** Farzad Hadaegh, Mitra Hasheminia, Hengameh Abdi, Davood Khalili, Mohammadreza Bozorgmanesh, Banafsheh Arshi, Fereidoun Azizi

**Affiliations:** 1 Prevention of Metabolic Disorders Research Center, Research Institute for Endocrine Sciences, Shahid Beheshti University of Medical Sciences, Tehran, Iran; 2 Endocrine Research Center, Research Institute for Endocrine Sciences, Shahid Beheshti University of Medical Sciences, Tehran, Iran; University of Utah School of Medicine, UNITED STATES

## Abstract

**Objective:**

Prehypertension is associated with cardiovascular disease (CVD). There is no study to examine the incidence and risk factors of prehypertension in a sex stratified setting. The aim of this study was to examine the effect modification of sex for different risk factors which predicts the progression from normotension to prehypertension in a Middle East population-based cohort, during a median follow-up of 9.2 years.

**Methods:**

A multivariate Cox analysis was performed among 1466 and 2131 Iranian men and women, respectively, who were free of prehypertension, hypertension, CVD and diabetes at baseline and free of incident hypertension without preceding prehypertension at follow-up. Incident prehypertension at follow-up was defined as systolic blood pressure (SBP) of 120–139 mmHg and/or diastolic blood pressure (DBP) of 80–89 mmHg.

**Results:**

Overall, 1440 new cases of prehypertension were identified resulting in an incidence rate of 593/10000 person-years; the corresponding values for women and men were 489/10000 and 764/10000person-years, respectively. There were significant interactions between gender with age, DBP, waist-to-hip-ratio (WHpR) and estimated glomerular filtration rate (eGFR) (all P-values <0.05) in multivariate analysis. Strong associations were found between age, body mass index (BMI) and SBP with incident prehypertension in both genders. However, the effect of DBP and WHpR was significant among women and 2-hour post challenge plasma glucose (2h-PCPG)was an independent risk factor for men. In the sex-adjusted analysis, glomerular hyperfiltration [Hazard ratio (HR) and 95%CI: 1.01 (1.00–1.01), P-value = 0.02], age, BMI, WHpR, SBP and DBP had higher risks while being female [HR (95%CI): 0.81(0.69–0.94), P-value = 0.01] had a lower risk for incident prehypertension.

**Conclusion:**

According to this study results, among Iranian population with high incidence of prehypertension, general adiposity and glomerular hyperfiltration in total, 2h-PCPG in men and central adiposity in women should be emphasized as risk factors for prehypertension.

## Introduction

In 2003, the Joint National Committee 7 (JNC7) proposed a new blood pressure category of 120 to 139 mm Hg systolic blood pressure (SBP) or 80 to 89 mm Hg diastolic blood pressure (DBP) and delegated it as prehypertension [1]. Prehypertension is associated with other cardiovascular risk factors such as obesity, insulin resistance, diabetes mellitus and dyslipidemia resulting in progressive atherosclerosis which can lead to cardiovascular disease (CVD) [2].

Recently, a meta-analysis with data from 468, 561 individuals in 18 prospective cohort studies showed that prehypertension elevated the risk of CVD, coronary heart disease and stroke [3]. Also, high normal blood pressure has been shown to be a risk factor for incident CVD only among middle-aged Iranian population [4]. On the other hand, it was recently shown that prehypertension is associated with CVD mortality, especially with stroke mortality, but not with all-cause mortality [5]. While the role of prehypertension itself as an independent risk factor for CVD and mortality events has been investigated, prospective population-based studies of the risk factors for prehypertension or its consequences are scarce [6].

Importantly, the development and progression of hypertension differs betweenmen and women. In fact,fundamental sex-related differences were reported in the mechanisms regulating blood pressure (i.e. in the renin—angiotensin system and sympathetic nervous system)[7]. There are well-established sex-related differences in the relationships between aging and arterial pressure. Pausova et al. have shown sex-related differences in the relationship between the distribution of body fat and arterial pressure in adolescence [8]. In a population-based study, a strong positive relationship was observed between arterial pressure and visceral fat in boys. However, in adolescent girls, arterial pressure was more strongly linked with total body fat rather than visceral fat [8]. Hence, as acknowledged by Denton et al [7],sexual dimorphism and the impact of sex steroids on genetic, hormonal and biochemical pathways that form cardiovascular function in men and women can be a reason to study cardiovascular risk factors in both genders, separately.

Although, many prospective studies examined the transition between normotension and hypertension [9],to the best of our knowledge, there is no study to examine the incidence and risk factors of prehypertension in a sex stratified setting. Moreover, the Middle East countries including Iran suffer from high prevalence of CVD risk factors including diabetes, hypertension, dyslipidemia and obesity [10]. A national surveillance conducted in 2007 in Iran showed a 26.6% prevalence rate for hypertension among Iranian adults [11]. The aim of this study was to explore sex-specific predictors of the progression from normotension to prehypertension in a Middle East population-based cohort, the Tehran Lipid and Glucose Study (TLGS).

## Methods

### Study population

TLGS is a prospective population-based study conducted on a representative sample of Tehranian population with the aim of determining the prevalence of non-communicable disease risk factors and developing a healthy lifestyle. Age distribution of the TLGS population at baseline is representative of the overall population of Tehran (Iran National Census, 1996). Data collection is ongoing, designed to continue for at least 20 years with follow-up examinations at about 3-year intervals [12]. Details of the study methods including the recruitment of participants, documentation of medical history and demographic data, clinical examinations, blood sample collections and laboratory and biochemical measurements are explained elsewhere, all of which follow the same method in every phase of TLGS [12].

**Fig 1:**
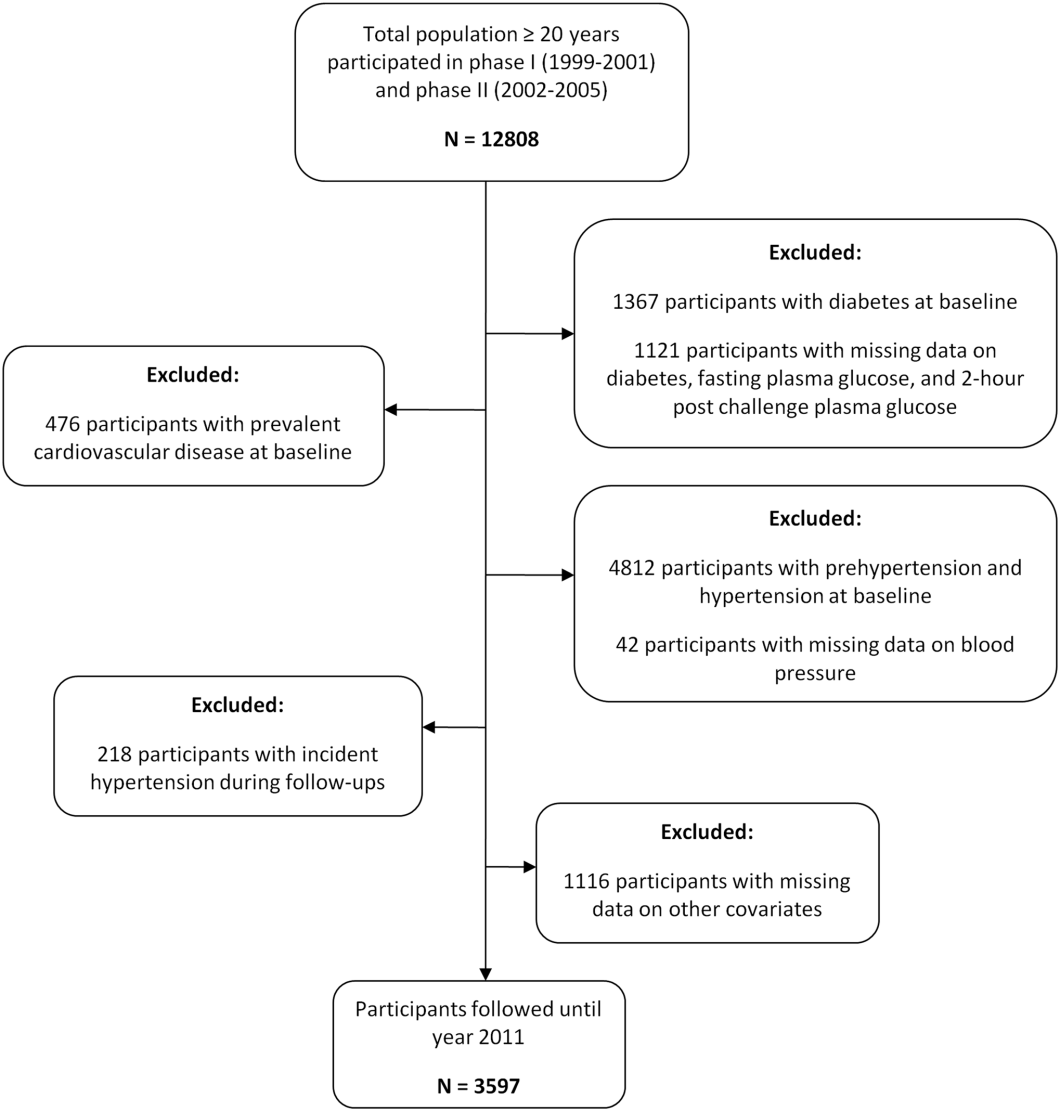
Study population selection flowchart in Tehran Lipid and Glucose Study (TLGS), 2001–2011.

To date, 4 phases with 18555 participants aged ≥3 years from district 13 of Tehran consisting of 15,005 first phase (1999–2001) and 3550 second phase recruitments (2002–2005) have been conducted. For the current study, 12,808 participants aged ≥20 years at baseline who were recruited from the first and second phase of TLGS were selected. Participants with prevalent diabetes or missing data of fasting plasma glucose (FPG) or 2-hour post-challenge plasma glucose (2h-PCPG) test (N = 2497) and prevalent CVD (n = 476) were excluded. Other exclusions included baseline prehypertension, baseline hypertension and missing data on hypertension status and those with incident hypertension without preceding prehypertension at follow-up (n = 5072). Finally, 1166 participants without any follow-up data or with missing data on covariates (non-respondents) out of the remaining 4763 were also removed from the study population which resulted in a final number of 3597 participants (1466 men and 2131 women) who were followed until 2011 (Fig 1). Hence, 75.5% of eligible baseline participants (3597/4763) were included in the current study. Among this population, serum fasting insulin data was available in 2114 participants (819 men and 1295 women).

Informed written consent was obtained from all participants and the Ethical Committee of Research Institute for Endocrine Sciences approved this study.

### Clinical and laboratory measurements

A trained interviewer collected information including demographic data, drug history, past medical history of CVD, diabetes and smoking status using a standard questionnaire [12]. Details of the anthropometric measurements including weight, height, waist circumference (WC) and hip circumference were reported elsewhere [12]. Body mass index (BMI) was calculated as weight in kilograms divided by height in square meters. After a 15-minute rest in the sitting position, systolic and diastolic blood pressures were measured twiceby atrained personnel, on the right arm, using a standardized mercury sphygmomanometer (calibrated by the Iranian Institute of Standards and Industrial Researches); the mean of the two measurements was considered as the participant’s blood pressure.

A blood sample was taken between 7:00 and 9:00 AM from all study participants, after 12 to 14 hours overnight fast. All blood analyses were carried out at the TLGS research laboratory on the day of blood sample collection. Details of laboratory measurements including FPG, 2h-PCPG test, total cholesterol (TC),triglycerides(TG), serum creatinine (Cr) levels and serum fasting insulin levels and the inter- and intra-assay coefficient of variables (CV) of these measurements were reported elsewhere [12, 13].

Physical activity level was assessed with the Lipid Research Clinic (LRC) questionnaire in the first baseline examination of the TLGS. Due to the impreciseness of LRCquestionnaire, it was substituted by the Modifiable Activity Questionnaire (MAQ) in the follow-up examinations. This questionnaire measures all three forms of activities including leisure time, job and household activities in the preceding year [12].

### Definition of terms

Hypertension at baseline and follow-ups was defined as the SBP ≥140 mmHg or DBP ≥90mmHg, or taking antihypertensive medication(s). Prehypertension at baseline and follow-ups was defined as the SBP ≥120 mmHg and <140mmHg and DBP ≥80 mmHg and <90mmHg [1].

For this study GFR was estimated using the abbreviated prediction equation, provided by the Modification of Diet in Renal Disease (MDRD) study as follows:

Abbreviated MDRD study equation:

$$\text{GFR}\;=\;186\;\times \;{\left(\text{SCr}\right)}^{-1.145}\;\times \;{\left(\text{Age}\right)}^{-0.203}\;\times \;\left(0.742\;\text{if}\;\text{female}\right)\;\times \;\left(1.210\;\text{if}\;\text{African}-\text{American}\right)$$

In this equation, estimated GFR (eGFR) was expressed as mL/min per 1.73 m^2^ and serum creatinine (Scr) was expressed as mg/dL [14]. For further analysis, eGFR was divided into quartiles in men (eGFR<69.34, 69.34≤eGFR<76.31, 76.31≤eGFR<84.42, eGFR ≥84.42),in women (eGFR<65.27, 65.27≤eGFR<73.01, 73.01≤eGFR<80.72, eGFR ≥80.72) and in the total population (eGFR<67.03, 67.03≤eGFR<74.31, 74.31≤eGFR<82.23, eGFR ≥82.23).

In accordance with the definition provided by the American Diabetes Association, participants were considered to have diabetes if they met at least one of these criteria: FPG ≥7 mmol/L, or 2h-PCPG ≥11.1 mmol/L or taking anti-diabetic medication [15]. Dyslipidemia was defined as TG ≥1.69 mmol/L or total cholesterol ≥6.21 mmol/L [16] or HDL-C <1.06 mmol/L (men) or HDL-C <1.29 mmol/L (women)[17] or using lipid lowering medications. Smoking status was categorized as current smokers (subjects who smoked cigarettes daily or occasionally), past smokers and never smokers. A previous history of CVD reflected any prior diagnosis of CVD by a physician. Individuals participating in a vigorous physical activity at least three days per week were categorized as physically active. Those participants who entered in the second phase, were considered physically active when achieving a minimum of at least 600 METs (metabolic equivalent task)-minutes per week [18]. Education was classified into 3 groups: Illiterate/primary school, below diploma/diploma and higher than diploma. Marital status was categorized into two groups: married versus widowed/divorced/single.

Insulin resistance (IR) was also estimated by the homeostasis model assessment (HOMA) of IR according to the formula [19]: HOMA-IR = [(Fasting insulin level (μU/mL) × FPG (mmol/L)]/ 22.5. HOMA-IR was divided into quartiles in men (HOMA-IR <0.9, 0.9 ≤ HOMA-IR <1.3, 1.3 ≤ HOMA-IR <1.9, and HOMA-IR ≥1.9) and women (HOMA-IR <1.1, 1.1≤HOMA-IR<1.6, 1.6 ≤ HOMA-IR <2.2, and HOMA-IR ≥2.2) and in the whole population (HOMA-IR <1.0, 1.0 ≤ HOMA-IR< 1.5, 1.5≤ HOMA-IR <2.1, and HOMA-IR ≥2.1) and modeled both as a continuous and categorical variable.

### Statistical analysis

Baseline characteristics of respondents and non-respondents (those without any follow-up data or missing data) were shown as mean (SD) or median (interquartile) and frequency (%) as appropriate. Comparisons between respondents and non-respondents were performed using Student’s T-test or Mann-Whitney test and χ2 tests as appropriate. Cumulative incidence of prehypertension with 95% (CI) was calculated for each sex by dividing the number of new cases of prehypertension to the total number of subjects. The annualized incidence rate of prehypertension was also calculated by dividing the total number of incident cases to the sum of person-times of follow-up with 95% confidence interval.

Univariable Cox analyses were performed for each potential risk factor including: Age (years), FPG (mmol/L), 2h-PCPG (mmol/L), BMI (kg/m^2^), WHpR, SBP (mmHg), DBP (mmHg), dyslipidemia (yes/no), eGFR (ml/min/1.73 m^2^), smoking status (never as reference, past, current), physically active (yes/no), education level (higher than diploma as reference, diploma/below diploma, illiterate/primary school), marital status (married as reference versus divorced/widowed or single) in each gender and whole population. Quartiles of eGFR were also introduced to the analysis to examine the association between eGFR and prehypertension. Then, covariates with a P-value less than 0.20 in the univariable analyses were selected to enter the multivariable model.

Cox proportional hazards models were used to evaluate associations of potential risk factors with incidence of prehypertension in men and women separately and also in the whole population. The event date for prehypertension cases was described as the mid-time between the date of follow-up visit at which prehypertension was detected for the first time, and the most recent follow-up visit preceding the diagnosis; the follow-up time was drawn from the difference between the calculated mid-time date and the date at which the subjects entered the study. For the censored participants, survival time was calculated as the interval between the first and the last observation dates. Follow-up duration and person-years were calculated using the measured survival time. Furthermore, to examine the independent role of insulin resistance besides other risk factors for incident prehypertension we repeated the multivariate analyses in each gender and the whole population in those with complete data for baseline insulin, considering the lowest quartile of HOMA-IR as reference.

In addition, we investigated whether sex modified the relations between potential risk factors and incidence of prehypertension. These analyses were performed by introducing interaction terms of risk factors with sex in the multivariable model. There were significant effect modifications of sex with age (P<0.001), DBP (P = 0.002), WHpR (P = 0.006), eGFR (P = 0.023) in multivariate analysis in the whole population. Hence, we stratified our analysis by sex. Finally, to be comparable with other studies, multivariable analyses were also carried out in a pooled data of both sexes, regardless of the significant interaction between sex and other risk factors.

The proportional hazard assumption of the multivariable Cox model was assessed using Schoenfeld’s global test of residuals. All analyses were performed using SPSS for Windows version 15 and STATA version 12 SE (Stata Corp LP, TX, USA), with a two-tailed P-value < 0.05 considered significant.

## Results

Table 1 shows baseline characteristics of the respondent and non-respondent men and women. Among women, respondents were older, had lower eGFR, higher FPG and SBP compared with non-respondents. Among men, respondents also had lower eGFR, but higher BMI, WHpR, TG and TC than non-respondents. Moreover, significant difference existed between respondents and non-respondents regarding history of smoking and marital status in both genders. Comparison between respondent men and women showed that men had higher age, WHpR, FPG, eGFR, TG, SBP, and lower BMI, HDL-C, 2h-PCPG and HOMA-IR level than women. Also, there was significant difference between two genders regarding education level and marital status.

**Table 1:** Comparison of baseline characteristics between the respondent versus non-respondent participants. Tehran Lipid and Glucose Study (TLGS), 2001–2011.

		Men (n = 1914)			Women (n = 2849)		
Variables	Respondent (N = 1466)	Non-Respondent (N = 448)	P-value*	Respondent (N = 2131)	Non-Respondent (N = 718)	P-value*	P-value**
Age(years)	38.10±12.11	36.89±11.78	0.06	34.62±10.05	32.81±10.48	< 0.001	< 0.001
SBP (mmHg)	106.7±7.59	106.4±8.03	0.41	104.4±8.03	103.4±8.14	0.005	< 0.001
DPB (mmHg)	69.43±6.26	69.42±6.47	0.99	69.20±6.27	68.81±6.81	0.16	0.28
BMI(kg/m^2^)	24.51±3.84	23.96±3.69	0.007	25.74±4.45	25.61±4.74	0.53	< 0.001
WHpR	0.90±0.07	0.89±0.07	0.009	0.81±0.07	0.81±0.07	0.87	< 0.001
FPG(mmol /l)	4.92±0.46	4.95±0.49	0.31	4.83±0.47	4.75±0.46	< 0.001	< 0.001
2h-PCPG (mmol /l)	5.26±1.45	5.26±1.43	0.97	5.61±1.35	5.60±1.34	0.86	< 0.001
eGFR(ml/min/1.73 m^2^)	77.32±11.42	78.61±11.28	0.04	73.39±11.25	76.46±12.69	< 0.001	< 0.001
Cholesterol (mmol /l)	4.99±1.08	4.83±1.02	0.005	4.96±1.09	4.91±1.15	0.24	0.47
TG (mmol /l)	1.47(1.04–2.17)	1.40(0.98–2.03)	0.05	1.16(0.85–1.70)	1.15(0.82–1.72)	0.53	< 0.001
HDL-C (mmol /l)	0.99±0.24	0.99±0.25	0.94	1.17±0.28	1.18±0.29	0.6	< 0.001
Lipid drug (%)	9(0.6%)	3(0.7%)	1.00	26(1.2%)	5(0.7%)	0.30	0.08
Dyslipidemia(%)^†^	1102(75.2%)	324(72.5%)	0.26	1644(77.1%)	560(78.2%)	0.57	0.17
HOMA-IR^‡^	1.34(0.92–1.88)	1.35(0.89–1.93)	0.93	1.57(1.13–2.18)	1.60(1.23–2.46)	0.30	< 0.001
Education Level(%)			0.67			0.12	< 0.001
Illiterate/ primary school	250(17.1%)	83(18.6%)		432(20.3%)	129(18%)		
Diploma/ below Diploma	916(62.5%)	279(62.4%)		1380(64.8%)	460(64.2%)		
Higher than diploma	300(20.5%)	85(19%)		319(15%)	128(17.9%)		
Smoking (%)			0.003			0.003	< 0.001
Never	795(54.2%)	204(45.7%)		2032(95.4%)	657(92%)		
Past	180(12.3%)	55(12.3%)		27(1.3%)	13(1.8%)		
Current	491(33.5%)	187(41.9%)		72(3.4%)	44(6.2%)		
Marital Status (%)			0.008			< 0.001	0.004
Married	1138(77.6%)	319(71.4%)		1739(81.6%)	532(74.1%)		
Divorced /widowed/Single	328(22.4%)	128(28.6%)		392(18.4%)	186(25.9%)		

Mean ± SD and median (Inter-quartile range) are shown for continuous variables. BMI: body mass index; WHpR: waist-to-hip-ratio; FPG: fasting plasma glucose; 2h-PCPG: 2-hr post challenge plasma glucose; SBP: systolic blood pressure; DBP: diastolic blood pressure; eGFR: estimated glomerular filtration rate; TC: total cholesterol; TG: triglycerides; HDL-C: High density lipoprotein cholesterol; HOMA-IR: Homeostasis model assessment of insulin resistance. The important findings of Table: Among women, respondents were older, had lower eGFR, higher FPG and SBP compared with non-respondents. Among men, respondents also had lower eGFR, but higher BMI, WHpR, TG and TC than non-respondents. Comparison between respondent men and women showed that men had higher age, WHpR, FPG, eGFR, TG, SBP, and lower BMI, HDL-C, 2h-PCPG and HOMA-IR level than women.

*P-value of the comparison between respondents and non-respondents in men and women, separately.

**P-value of the comparison between respondent men and women.

^†^ Dyslipidemia was defined as TG ≥ 1.69 mmol/L or total cholesterol ≥ 6.21 mmol/L or HDL-C < 1.06 mmol/L (men) or HDL-C<1.29 mmol/L (women) or using lipid lowering medications.

^‡^ Insulin data was available in 2114 respondent (men:819, women: 1295)and 79 non-respondent (men:8, women: 71)population.

Comparison between baseline characteristics of participants with and without incident prehypertension are illustrated in Table 2. As shown, in both genders, participants who developed incident prehypertension were older, had higher BMI, WHpR, FPG, 2h-PCPG, SBP, DBP, TG, TC and HOMA-IR but lower eGFR. Moreover, women with incident prehypertension had lower HDL-C than the non-incident group. Overall, 1440 new cases of prehypertension (735 women and 705 men) were identified after a median follow-up of 9.2 years (interquartile range 6.8 to 10.4 years) resulting in an incidence rate of 593/10000 person-years (95% CI: 564–625). The incidence rate of prehypertension among women [489/10000 person-years (95% CI: 455–526)] was significantly lower than men [764/10000person-years, (95% CI: 709–822)], (P<0001). Additionally, we observed that during the study follow-up untilphase 3 TLGS (i.e. 2005–2008) with a median follow-up of 5.9years, 872(432 women and 440 men) new events of prehypertension occurred resulting in an incidence rate of 546/10000 person-years (95% CI: 511–584); the corresponding values for women and men were 443/10000 (95% CI: 404–487) and 715/10000 person-years (95% CI: 651–786), respectively.

**Table 2:** Comparison of baseline characteristics of participants with and without incident prehypertension during 9.2 years follow-up. Tehran Lipid and Glucose Study (TLGS), 2001–2011.

		Men (n = 1466)			Women (n = 2131)	
Variables	With incident Prehypertension (n = 705)	Without incident Prehypertension (n = 761)	P-value*	With incident Prehypertension (n = 735)	Without incident Prehypertension (n = 1396)	P-value*
Age(years)	39.61±12.50	36.7±11.57	< 0.001	38.30±10.56	32.68±9.20	< 0.001
SBP (mmHg)	108.6±7.0	105.0±7.70	< 0.001	107.1±7.15	103.0±8.11	< 0.001
DPB (mmHg)	70.46±5.79	68.47±6.53	< 0.001	71.45±5.37	68.01±6.39	< 0.001
BMI(kg/m^2^)	25.22±3.90	23.86±3.68	< 0.001	27.17±4.69	24.99±4.13	< 0.001
WHpR	0.91±0.07	0.89±0.06	< 0.001	0.83±0.08	0.80±0.07	< 0.001
FPG (mmol/l)	4.96±0.48	4.89±0.44	< 0.001	4.92±0.5	4.78±0.44	< 0.001
2h-PCPG (mmol/l)	5.43±1.53	5.11±1.35	< 0.001	5.90±1.47	5.46±1.26	< 0.001
eGFR(ml/min/1.73 m^2^)	76.50±11.40	78.09±11.40	0.008	71.49±11.65	74.40±10.91	< 0.001
Cholesterol (mmol/l)	5.14±1.03	4.85±1.11	< 0.001	5.19±1.18	4.85±1.02	< 0.001
TG (mmol /l)	1.64(1.11–2.37)	1.36(0.98–2.03)	< 0.001	1.33(0.94–2.03)	1.08(0.8–1.53)	< 0.001
HDL-C (mmol /l)	0.98±0.25	0.99±0.24	0.57	1.15±0.29	1.18±0.28	0.02
Lipid drug (%)	4(0.6%)	5(0.7%)	1	17(2.3%)	9(0.6%)	0.001
Dyslipidemia (%)**	548(77.7%)	554(72.8%)	0.03	595(81%)	1049(75.1%)	0.002
HOMA-IR†	1.41(0.97–2.00)	1.25(0.87–1.81)	0.02	1.68(1.23–2.26)	1.51(1.1–2.15)	0.001
Education Level (%)			0.02			< 0.001
Illiterate/ primary school	140(19.9%)	110(14.5%)		210(28.6%)	222(15.9%)	
Diploma/ below Diploma	427(60.6%)	489(64.3%)		434(59%)	946(67.8%)	
Higher than diploma	138(19.6%)	162(21.3%)		91(12.4%)	228(16.3%)	
Smoking (%)			0.34			0.96
Never	392(55.6%)	403(53%)		700(95.2%)	1332(95.4%)	
Past	90(12.8%)	90(11.8%)		10(1.4%)	17(1.2%)	
Current	223(31.6%)	268(35.2%)		25(3.4%)	47(3.4%)	
Marital Status (%)			< 0.001			< 0.001
Married	576(81.7%)	562(73.9%)		633(86.1%)	1106(79.2%)	
Divorced /widowed/Single	129(18.3%)	199(26.1%)		102(13.9%)	290(20.8%)	

Mean ± SD and median (Inter-quartile range) are shown for continuous variables. BMI: body mass index; WHpR: waist-to-hip-ratio; FPG: fasting plasma glucose; 2h-PCPG: 2-hr post challenge plasma glucose; SBP: systolic blood pressure; DBP: diastolic blood pressure; eGFR: estimated glomerular filtration rate; TC: total cholesterol; TG: triglycerides; HDL-C: High density lipoprotein cholesterol; HOMA-IR: Homeostasis model assessment of insulin resistance. The important findings of Table: In both genders, participants who developed incident prehypertension were older, had higher BMI, WHpR, FPG, 2h-PCPG, SBP, DBP, TG, TC and HOMA-IR but lower eGFR.

*P-value of the comparison between the incident and censored participants in each gender.

** Dyslipidemia was defined as TG ≥ 1.69 mmol/L or total cholesterol ≥ 6.21 mmol/L or HDL-C < 1.06 mmol/L (men) or HDL-C<1.29 mmol/L (women) or using lipid lowering medications.

^†^ Insulin data was available in 2114 participants (men: 819, women: 1295).

Table 3 and S1 Table show the adjusted hazard ratios (HRs) and 95% confidence intervals (95%CIs) of prehypertension associated with baseline risk factors in each gender and the whole population in models with and without HOMA-IR, respectively. Age, BMI and SBP were significant predictors of prehypertension in each gender and also in the sex-adjusted analysis. Furthermore as shown in Table 3, 2h-PCPG was an independent predictor only in men [HR (95% CI): 1.06(1.01–1.12)], whileWHpR and DBP were the significant predictors only in women [HRs (95%CIs): 1.24(1.11–1.39) and 1.04(1.03–1.06), respectively]. Also, in the sex-adjusted model, female gender introduced lower risk for incident prehypertension [HR (95% CI): 0.81(0.69–0.94)] and increase in eGFR resulted in significant risk of prehypertension. Among participants with baseline insulin data, HOMA-IR did not show any risk for incident prehypertension (S1 Table). Finally, when we categorized eGFR into quartiles, the 4th quartile of eGFR (i.e. eGFR ≥82.23 ml/min/1.73 m^2^) was associated with 21% increased risk of prehypertension in multivariate sex-adjusted analysis (S2 Table).

**Table 3:** Multivariable adjusted Hazard ratios (HRs [95% CIs]) of predictors of incident prehypertension. Tehran Lipid and Glucose Study (TLGS), 2001–2011.

	Men (n = 1466)		Women (n = 2131)		Total population (n = 3597)	
Variables	HRs(CI)	P-value	HRs(CI)	P-value	HRs(CI)	P-value
Age(years)	1.01 (1.00–1.02)	0.01	1.04(1.03–1.05)	<0.001	1.02 (1.02–1.03)	<0.001
Gender (Female)	—	—	—	—	0.81(0.69–0.94)	0.01
SBP(mmHg)	1.05(1.04–1.06)	<0.001	1.03(1.02–1.04)	<0.001	1.04(1.03–1.05)	<0.001
DPB(mmHg)	1.01(0.99–1.02)	0.22	1.04 (1.03–1.06)	<0.001	1.03(1.02–1.04)	<0.001
BMI (kg/ m^2^)	1.04(1.01–1.07)	0.01	1.04 (1.02–1.06)	<0.001	1.04(1.03–1.06)	<0.001
WHpR	1.11(0.94–1.32)	0.22	1.24 (1.11–1.39)	<0.001	1.18(1.08–1.30)	<0.001
FPG (mmol/l)	1.01(0.86–1.19)	0.91	1.10(0.93–1.30)	0.26	1.05(0.93–1.18)	0.42
2h-PCPG (mmol/l)	1.06(1.01–1.12)	0.03	1.01(0.95–1.07)	0.84	1.04(0.99–1.08)	0.09
eGFR(ml/min/1.73m^2^)	1.01(1.00–1.02)	0.07	1.01(1.00–1.02)	0.08	1.01(1.00–1.01)	0.02
Dyslipidemia*	1.04 (0.87–1.26)	0.65	0.98 (0.81–1.19)	0.86	1.00(0.87–1.14)	0.97
Education Level						
Higher than Diploma	Reference	-	Reference	-	Reference	-
Diploma/Below Diploma	1.08(0.89–1.31)	0.44	0.94(0.75–1.18)	0.59	1.04(0.90–1.20)	0.62
Illiterate/PrimarySchool	1.28(0.99–1.65)	0.05	0.87 (0.67–1.15)	0.33	1.11(0.92–1.33)	0.28
Smoking						
Never	—	—	—	—	Reference	-
Past	—	—	—	—	1.02 (0.82–1.27)	0.83
Current	—	—	—	—	1.01 (0.87–1.18)	0.87
Marital status						
Married	Reference	-	Reference	-	Reference	-
Divorced/Widowed/Single	0.91 (0.73–1.14)	0.41	0.96(0.78–1.19)	0.74	1.02(0.87–1.18)	0.84

Cox proportional hazard models were used to calculate HRs and 95% CIs. eGFR: estimated glomerular filtration rate; FPG: fasting plasma glucose; TG: triglycerides; HDL-C: High density lipoprotein cholesterol; SBP: systolic blood pressure; DBP: diastolic blood pressure; TC: total cholesterol; BMI: body mass index; WHpR: waist-to-hip-ratio; 2h-PCPG: 2-h post challenge plasma glucose. The important findings of Table: Age, BMI and SBP were significant predictors of prehypertension in each gender and also in the sex adjusted analysis.2h-PCPG was an independent predictor only in men, while WHpR and DBP were the significant predictors only in women. In the sex adjusted model, female gender had lower risk for incident prehypertension than male andincrease in eGFR resulted in significant risk of prehypertension.

*Dyslipidemia was defined as TG ≥ 1.69 mmol/L or total cholesterol ≥ 6.21 mmol/L or HDL-C < 1.06 mmol/L (men) or HDL-C<1.29 mmol/L (women) or using lipid lowering medications.

## Discussion

To the best of our knowledge, the current study is the first prospective population-based study to examine the sex-specific incidence of prehypertension. In the present survey, among Iranian adult men and women, during 9.2 years of follow-up, the incidence rates of prehypertension were 489/10000 person-years (95% CI: 455–526) in women and 764/10000 person-years (95% CI: 709–822) in men. Furthermore, the role of different risk factors in progression to prehypertension was investigated. Our study demonstrated strong association between age, BMI and SBP with prehypertension development in both sexes. However, the effect of DBP and WHpR was only significant among women and 2h-PCPG was an independent risk factor only in men. In the sex-adjusted analysis, the male gender and higher values of eGFR, BMI, WHpR, SBP and DBP were associated with incident prehypertension.

The incidence of prehypertension in this population is higher than some other reports. The sex-pooled incidence of prehypertension was reported to be 37% in China [20], 33% in the Western New York Health Study [6] and 46% in Japan [21]. The differences observed in the incidence of prehypertension between different populations might be related to the variation in ethnicity and different population characteristics [22]. As a result, the reported high incidence of prehypertension in the current study might be attributable to the alarming rise of obesity [23], increasing trend of diabetes incidence [24] as the coexistent cardiovascular risk factors with prehypertension [25–27], high prevalence of low physical activity (40% in a national survey) [28]and unhealthy diet specifically high amount of dietary salt intake [29], in Iran. In a study by Khosravi et al. on a representative adult population in Isfahan province, it was shown that the amount of urinary excretion of sodium was more than 8 gr/day; hence, it was estimated that the equivalent salt intake was between 9 to 11.8 gr/day [30]. Regarding psychosocial characteristics, in a recent study in south of Iran, among 5900 participants aged 15–75 years, the prevalence of prehypertension was reported to be 35.5%. The men had higher rate of prehypertension (42.7 vs. 28.1%) than did women. The study showed that besides other traditional risk factors, anxiety which was found to have a prevalence of about 50% among normotensive population, remained as a significant risk factor for hypertension but not for prehypertension [31].

Regarding impact modification of sex for risk factors of incident prehypertension,we found that the association between age, WHpR and DBP and incident prehypertension was stronger among women compared to men. This difference might be related to sex hormones [32]. On the other hand, the pooled incidence rate of prehypertension was considerably higher among men. Markedly, women had about 19% lower risk for incident prehypertension than men in the multivariate analysis. The higher risk of prehypertension among men is supported by many cross-sectional studies showing that male gender has a stronger association with prevalent hypertension [2, 33], indicating the probable presence of complex interaction of mechanisms. Similar findings have been reported in a recent meta-analysis of epidemiology and risk factors of prehypertension [32]. In fact, the results of our study underscores the higher risk of another CVD risk factor, i.e. prehypertension among men.

Prehypertension and hypertension share the same risk factors, such as obesity, type 2 diabetes and lipid disorders [34]. Although in our general population, indices of general obesity were not determinants of incident hypertension [35], we found these measures as risk factors for incident prehypertension. Therefore, it is necessary to differentiate the risk factors of incident hypertension and prehypertension. In accordance with the results of some other studies [2, 36, 37], we demonstrated a strong association between BMI and WHpR with prehypertension. In a cross- sectional survey in Taiwan [36], it was shown that general adiposity was significantly associated with prehypertension in men. Moreover, central adiposity, measured by WC, was an important determinant only in women. We recently found that the prevalence of general and central adiposity among non-diabetic Iranian population increased significantly during 10 years of follow-up which could lead to high incidence of prehypertension among Tehranian adult population [38]. Nevertheless, the precise mechanism through which general and or central adiposity increases blood pressure is not entirely understood. Importantly, the significant risk of general adiposity in both genders and central adiposity among women in our study was shown in the presence of insulin resistance. Also, clinical studies have shown that weight loss is the most effective lifestyle modification strategy for prevention of hypertension [39].

In a 12 year follow-up study of American Indians, the prevalence of prehypertension was significantly higher in those with incident diabetes at follow-up than in those who did not [40]. It has also been shown that individuals with prehypertension are more likely to have diabetes [41] and impaired fasting glucose than normotensive individuals [2]. In the Western New York Health Study, the presence of impaired fasting glucose (IFG) at baseline and weight gain were the strongest significant predictors of prehypertension at follow-up; however, the researchers did not examine the impact of IFG in the presence of impaired glucose tolerance or insulin resistance [6]. We recently found that insulin resistance was an independent risk factor for developing hypertension among women [13]. Although in the current study we did not find such relationship between insulin resistance and prehypertension in the whole population, our results revealed 2h-PCPG as an important risk factor for incident prehypertension among men.

Regarding kidney function, many studies have shown an independent association between hypertension incidence, consequent mortality events and lower eGFR levels [42–44]. In the present study, to examine the association between eGFR and prehypertension we applied a continuous rather than a categorical approach, as recently suggested by Cachat et al [45]. In addition, we showed that eGFR ≥82.23 ml/min/1.73 m^2^ was associated with more than 20% increase in risk of future prehypertension. The association between hypertension and the increased risk of glomerular hyperfiltration is not unexpected, as elevated blood pressure is a known driver of glomerular capillary hydraulic pressure and glomerular filtration [46]. There is no consensus on the definition of glomerular hyperfiltration and its pathophysiological mechanisms, which seem to differ with the underlying condition and are not well distinguished [45, 47]. Among overweight and obese populations, eGFR increased more than renal blood flow, leading to an increase in filtration fraction. The metabolic syndrome has also shown to be associated with glomerular hyperfiltration [47]. Additionally, higher levels of filtration fraction were reported in salt-sensitive individuals [48]. Therefore, it might be concluded that in an Iranian population with highly prevalent obesity [23], metabolic syndrome [17] and high salt intake [30], the glomerular hyperfiltration can be a risk factor for incident prehypertension. However, the mechanisms linking glomerular hyperfiltration to consequent prehypertension remain obscure.

Among socioeconomic factors, just lower rate of education had a marginally significant impact on the development of prehypertension only among men that agrees with the results of a national survey conducted among Iranian population [49]. We were surprised to find that certain variables did not independently predict the incident prehypertension after multivariate adjustment, including physical activity. Data from 2 combined non-concurrent cohort studies showed that exercise or physical activity significantly contributes to lowering the risk of hypertension [50]. Although performing regular physical activity is a scientifically supported protective factor for prevention of hypertension, it often fails to reach statistical significance due to various issues (e.g., difficulty in quantifying, measurement error or misclassification) among many studies [51]. On the other hand, the marital status of our study population did not affect the incidence of prehypertension. In contrast, among more than 17000 middle-aged Swedish participants in a prospective study, it was shown that being married or living as a couple significantly reduced the incidence of hypertension during follow-up[OR = 0.879 (0.802–0.964)][52].

The strengths of the present study could be the rational size of population, length of follow-up and using actual measurements of variables rather than self-reported data. Moreover, the sex specific method of this study adds to the understanding of different contribution of risk factors to the development of prehypertension in men and women, separately. However, several limitations of the current study need to be addressed. Firstly, some important risk factors for prehypertension such as nutritional data were not measured. Despite its importance, dietary factors are very difficult to measure with adequate precision. Secondly, this study has been conducted on a sample of Iranian population and further studies should be conducted to determine whether our findings can be applicable to other populations. The last but not least, although we found mild increase in the incidence of prehypertension through median follow-up of 5.9 to 9.2 years, because of some overlap in their 95% CIs and also the nature of our study as a fixed cohort, it was not possible for us to forecast the incidence of prehypertension, bearing in mind that in a fixed cohort it is not easy to discriminate age, period and cohort effects [53]. However, there is still potential use for predicting burden of prehypertension in the future. We observed the independent risk of aging in prediction of prehypertension in both genders. The importance of time will be much more evident when it is looked upon in the light of the fact that the life expectancy has increased during last decades [54]. The time trends in obesity, another predictor of prehypertension, have been shown to be increasing [38]. Thus, we can expect that, in the future, the instantaneous incidence rate of prehypertension will be larger than what we observed.

In summary, the findings of the present study showed that about half of Iranian population developed prehypertension during a decade follow-up which was more prominent among men. Sex significantly modified the impact of age, DBP, WHpR and eGFR for incident prehypertension in the whole population. We also observed that prehypertension incidence was strongly associated with age, BMI and SBP in both genders. However, WHpR and DBP only among women and 2h-PCPG only among men were related to incident prehypertension. Furthermore, in the sex-adjusted analysis glomerular hyperfiltration significantly increased the risk of subsequent prehypertension. Thus, emergent intervention is necessary to halt the tsunami of prehypertension among the Iranian population, considering specific risk factors in each gender.

## Supporting Information

S1 Table Multivariable adjusted hazard ratios [HRs (95% CIs)] of predictors of incident prehypertension including HOMA-IR. Tehran Lipid and Glucose Study (TLGS), 2001–2011.
(DOC)

S2 Table Multivariable adjusted hazard ratios [HRs (95% CIs)] of predictors for incident prehypertension including quartiles of eGFR. Tehran Lipid and Glucose Study (TLGS), 2001–2011.
(DOC)

S1 Dataset Baseline characteristics and the outcome of the study population, Tehran Lipid and Glucose Study (TLGS), 2001–2011. (XLS)
