# Supplementary material for: Prehypertension Tsunami: A Decade Follow-Up of an Iranian Adult Population
Source: PLoS One. 2015 Oct 6;10(10):e0139412. doi: 10.1371/journal.pone.0139412 (PMC4595371; doi:10.1371/journal.pone.0139412)
Supplement: S1 Table — Tehran Lipid and Glucose Study (TLGS), 2001–2011. (DOC) [file pone.0139412.s001.doc]

| **S1 Table. Multivariable adjusted hazard ratios [HRs (95% CIs)] of predictors of incident prehypertension including HOMA-IR. Tehran Lipid and Glucose Study (TLGS), 2001-2011** | | | | | | | | | |
| --- | --- | --- | --- | --- | --- | --- | --- | --- | --- |
|  |  | Men (n=819) | |  | Women (n=1295) | |  | Total population (n=2114)* | |
| Variables |  | HRs(CI) | P-value |  | HRs(CI) | P-value |  | HRs(CI) | P-value |
| Age(years) |  | 1.02(1.00-1.03) | 0.007 |  | 1.03 (1.02-1.05) | <0.001 |  | 1.02(1.02-1.03) | <0.001 |
| Gender(Female) |  |  |  |  |  |  |  | 0.75 (0.61-0.92) | 0.006 |
| SBP(mmHg) |  | 1.05(1.03-1.06) | <0.001 |  | 1.03(1.01-1.04) | <0.001 |  | 1.04(1.03-1.05) | <0.001 |
| DPB(mmHg) |  | 1.02(1.00-1.03) | 0.09 |  | 1.05 (1.03-1.07) | <0.001 |  | 1.03 (1.02-1.05) | <0.001 |
| BMI (kg/ m^2^) |  | 1.06 (1.02-1.10) | 0.001 |  | 1.03 (1.01-1.06) | 0.01 |  | 1.04 (1.02-1.06) | <0.001 |
| WHpR |  | 0.99(0.79-1.23) | 0.90 |  | 1.23 (1.06-1.43) | 0.005 |  | 1.13(1.00-1.27) | 0.049 |
| FPG (mmol/l) |  | 1.19 (0.94-1.52) | 0.15 |  | 1.02(0.81-1.27) | 0.89 |  | 1.09(0.93-1.28) | 0.31 |
| 2h-PCPG (mmol/l) |  | 1.12(1.04-1.20) | 0.003 |  | 1.01(0.93-1.09) | 0.89 |  | 1.06 (1.01-1.12) | 0.02 |
| eGFR(ml/min/1.73m^2^) |  | 1.01 (1.00-1.02) | 0.03 |  | 1.01(1.00-1.02) | 0.10 |  | 1.01(1.00-1.02) | 0.009 |
| Dyslipidemia** |  | 1.02 (0.81-1.30) | 0.84 |  | 1.00(0.79-1.27) | 1.00 |  | 1.00(0.85-1.19) | 0.97 |
| Education Level |  |  |  |  |  |  |  |  |  |
| Higher than Diploma |  | Reference | - |  | Reference | - |  | Reference | - |
| Diploma/ Below Diploma |  | 1.14(0.88-1.47) | 0.33 |  | 0.95(0.71-1.26) | 0.71 |  | 1.05(0.87-1.27) | 0.63 |
| Illiterate/Primary School |  | 1.25(0.89-1.76) | 0.20 |  | 0.88(0.63-1.24) | 0.47 |  | 1.09 (0.86-1.38) | 0.47 |
| Smoking |  |  |  |  |  |  |  |  |  |
| Never |  | Reference | - |  | Reference | - |  | Reference | - |
| Past |  | ـــــــــ | ـــــــــ |  | ـــــــــ | ـــــــــ |  | 1.14 (0.87-1.50) | 0.35 |
| Current |  | ـــــــــ | ـــــــــ |  | ـــــــــ | ـــــــــ |  | 1.05 (0.86-1.29) | 0.63 |
| Marital status |  |  |  |  |  |  |  |  |  |
| Married |  | Reference |  |  | Reference | - |  | Reference |  |
| Divorced/Widowed/Single |  | 0.94 (0.71-1.26) | 0.69 |  | 1.00(0.76-1.32) | 0.99 |  | 1.03(0.85-1.24) | 0.80 |
| HOMA-IR† |  |  |  |  |  |  |  |  |  |
| HOMA-IR Quartile (1) |  | Reference | - |  | Reference | - |  | Reference | - |
| HOMA-IR Quartile (2) |  | 0.87 (0.65-1.18) | 0.37 |  | 0.98(0.74-1.30) | 0.87 |  | 0.96 (0.79-1.18) | 0.72 |
| HOMA-IR Quartile (3) |  | 1.08(0.79-1.46) | 0.65 |  | 1.16(0.88-1.53) | 0.30 |  | 0.98 (0.80-1.21) | 0.88 |
| HOMA-IR Quartile (4) |  | 0.81(0.57-1.14) | 0.23 |  | 1.04(0.77-1.41) | 0.80 |  | 0.93 (0.74-1.17) | 0.53 |
| Cox proportional hazard models were used to calculate HRs and 95% CIs.  BMI: body mass index; WHpR: waist-to-hip-ratio; FPG: fasting plasma glucose; 2h-PCPG: 2-hr post challenge plasma glucose; SBP: systolic blood pressure; DBP: diastolic blood pressure; eGFR: estimated glomerular filtration rate; TC: total cholesterol; TG: triglycerides; HDL-C: High density lipoprotein cholesterol; HOMA-IR: Homeostasis model assessment of insulin resistance.; HOMA IR quartiles in men: HOMA-IR <0.9, 0.9 ≤ HOMA-IR <1.3, 1.3 ≤ HOMA-IR <1.9, and HOMA-IR ≥1.9; in women: HOMA-IR < 1.1, 1.1≤HOMA-IR< 1.6, 1.6 ≤ HOMA-IR < 2.2, and HOMA-IR ≥ 2.2; in the total population: HOMAIR <1.0, 1.0 ≤ HOMA-IR< 1.5, 1.5 ≤ HOMA-IR <2.1, and HOMA-IR ≥ 2.1  The HR for age, eGFR, BMI, WHpR, FPG, 2h-PCPG, SBP, DBP was continuous and calculated for 1 unit.  ** Dyslipidemia was defined as TG ≥ 1.69 mmol/L or total cholesterol ≥ 6.21 mmol/L or HDL-C < 1.06 mmol/L (men) or HDL-C<1.29 mmol/L (women) or using lipid lowering medications.  †Insulin data was available in 2114 participants (men: 819, women: 1295).*The important findings of Table.Among participants with baseline insulin data, HOMA-IR did not show any risk for incident prehypertension.* | | | | | | | | | |
