# Supplementary material for: Prehypertension Tsunami: A Decade Follow-Up of an Iranian Adult Population
Source: PLoS One. 2015 Oct 6;10(10):e0139412. doi: 10.1371/journal.pone.0139412 (PMC4595371; doi:10.1371/journal.pone.0139412)
Supplement: S2 Table — Tehran Lipid and Glucose Study (TLGS), 2001–2011. (DOC) [file pone.0139412.s002.doc]

| **S2 Table. Multivariable adjusted hazard ratios [HRs (95% CIs)] of predictors for incident prehypertension including quartiles of eGFR. Tehran Lipid and Glucose Study (TLGS), 2001-2011** | | | | | | | | | |
| --- | --- | --- | --- | --- | --- | --- | --- | --- | --- |
|  |  | Men (n=1466) | |  | Women (n=2131) | |  | Total population (n=3597) | |
| Variables |  | HRs(CI) | P-value |  | HRs(CI) | P-value |  | HRs(CI) | P-value |
| Age(years) |  | 1.01(1.00-1.02) | 0.02 |  | 1.03 (1.03-1.04) | <0.001 |  | 1.02 (1.02-1.03) | <0.001 |
| Gender (Female) |  | ـــــــــ | ـــــــــ |  | ـــــــــ | ـــــــــ |  | 0.80(0.69-0.94) | 0.006 |
| SBP (mmHg) |  | 1.05(1.04-1.06) | <0.001 |  | 1.03(1.02-1.04) | <0.001 |  | 1.04(1.03-1.05) | <0.001 |
| DPB (mmHg) |  | 1.01(0.99-1.02) | 0.24 |  | 1.04(1.03-1.06) | <0.001 |  | 1.03(1.01-1.04) | <0.001 |
| BMI (kg/ m^2^) |  | 1.04(1.01-1.07) | 0.01 |  | 1.04(1.02-1.06) | <0.001 |  | 1.04(1.03-1.06) | <0.001 |
| WHpR |  | 1.11(0.93-1.31) | 0.24 |  | 1.24(1.11-1.39) | <0.001 |  | 1.18(1.08-1.30) | <0.001 |
| FPG (mmol/l) |  | 1.01(0.86-1.20) | 0.88 |  | 1.20(0.93-1.29) | 0.27 |  | 1.05(0.93-1.18) | 0.42 |
| 2h-PCPG (mmol/l) |  | 1.06(1.00-1.12) | 0.03 |  | 1.01(0.95-1.07) | 0.83 |  | 1.03(0.99-1.08) | 0.10 |
| eGFR(ml/min/1.73m^2^)* |  |  |  |  |  |  |  |  |  |
| eGFR Quartile(1) |  | Reference | - |  | Reference | - |  | Reference | - |
| eGFR Quartile(2) |  | 1.06(0.86-1.31) | 0.58 |  | 1.02(0.83-1.25) | 0.87 |  | 0.99(0.86-1.15) | 0. 91 |
| eGFR Quartile(3) |  | 0.99(0.79-1.24) | 0.90 |  | 1.11(0.89-1.38) | 0.36 |  | 1.04(0.89-1.21) | 0.64 |
| eGFR Quartile(4) |  | 1.23(0.98-1.55) | 0.08 |  | 1.25(0.98-1.58) | 0.07 |  | 1.21(1.03-1.43) | 0.02 |
| Dyslipidemia** |  | 1.05(0.87-1.26) | 0.63 |  | 0.98(0.81-1.19) | 0.85 |  | 1.00(0.88-1.14) | 0.99 |
| Education Level |  |  |  |  |  |  |  |  |  |
| Higher than Diploma |  | Reference | - |  | Reference | - |  | Reference | - |
| Diploma/ Below Diploma |  | 1.08(0.89-1.31) | 0.45 |  | 0.94(0.75-1.18) | 0.58 |  | 1.04(0.90-1.21) | 0.58 |
| Illiterate/Primary School |  | 1.29(1.00-1.66) | 0.05 |  | 0.87(0.66-1.14) | 0.31 |  | 1.10 (0.92-1.33) | 0.29 |
| Smoking |  |  |  |  |  |  |  |  |  |
| Never |  | ـــــــــ | ـــــــــ |  | ـــــــــ | ـــــــــ |  | Reference | - |
| Past |  | ـــــــــ | ـــــــــ |  | ـــــــــ | ـــــــــ |  | 1.02 (0.82-1.27) | 0.83 |
| Current |  | ـــــــــ | ـــــــــ |  | ـــــــــ | ـــــــــ |  | 1.01 (0.87-1.18) | 0.86 |
| Marital status |  |  |  |  |  |  |  |  |  |
| Married |  | Reference | - |  | Reference | - |  | Reference | - |
| Divorced/Widowed/Single |  | 0.90(0.72-1.12) | 0.34 |  | 0.96(0.78-1.19) | 0.71 |  | 1.00(0.86-1.17) | 0.97 |
| Cox proportional hazard models were used to calculate HRs and 95% CIs. eGFR: estimated glomerular filtration rate; FPG: fasting plasma glucose; TG: triglycerides; HDL-C: High density lipoprotein cholesterol; SBP: systolic blood pressure; DBP: diastolic blood pressure; TC: total cholesterol; BMI: body mass index; WHpR: waist-to-hip-ratio; 2h-PCPG: 2-h post challenge plasma glucose.  The HR for age, eGFR, BMI, WHpR, FPG, 2h-PCPG, SBP, DBP was continuous and calculated for 1 unit.  *eGFR quariles in men : eGFR <69.34, 69.34≤ eGFR <76.31,76.31≤ eGFR <84.42,eGFR ≥84.42 ; in women : eGFR <65.27, 65.27≤ eGFR<73.01,73.01≤ eGFR <80.72,eGFR ≥80.72; in the total population: eGFR <67.03, 67.03≤ eGFR <74.31,74.31≤ eGFR <82.23,eGFR ≥82.23  ** Dyslipidemia was defined as TG ≥ 1.69 mmol/L or total cholesterol ≥ 6.21 mmol/L or HDL-C < 1.06 mmol/L (men) or HDL-C<1.29 mmol/L (women) or using lipid lowering medications.*The important findings of Table :the 4th quartile of eGFR (i.e. eGFR ≥82.23 ml/min/1.73 m2) was associated with 21% increased risk of prehypertension in multivariate sex-adjusted analysis* | | | | | | | | | |
